# Analyzing patient perspectives with large language models: a cross-sectional study of sentiment and thematic classification on exception from informed consent

**DOI:** 10.1038/s41598-025-89996-w

**Published:** 2025-02-20

**Authors:** Aaron E. Kornblith, Chandan Singh, Johanna C. Innes, Todd P. Chang, Kathleen M. Adelgais, Maija Holsti, Joy Kim, Bradford McClain, Daniel K. Nishijima, Steffanie Rodgers, Manish I. Shah, Harold K. Simon, John M. VanBuren, Caleb E. Ward, Catherine R. Counts

**Affiliations:** 1 University of California San Francisco, San Francisco, CA USA; 2 Microsoft Research, Seattle, WA USA; 3 Jacobs School of Medicine and Biomedical Sciences, University at Buffalo, Buffalo, NY USA; 4 Keck School of Medicine of University of Southern California & Children’s Hospital Los Angeles, Los Angeles, CA USA; 5 University of Colorado School of Medicine, Aurora, CO USA; 6 Primary Children’s Medical Center, University of Utah, Salt Lake City, UT USA; 7 Oregon Health & Science University, Portland, OR USA; 8 Cincinnati Children’s Hospital Medical Center, Cincinnati, OH USA; 9 UC Davis School of Medicine, Sacramento, CA USA; 10 Nationwide Children’s Hospital, Columbus, OH USA; 11 Stanford University School of Medicine, Palo Alto, CA USA; 12 Emory University School of Medicine & Children’s Healthcare of Atlanta, Atlanta, GA USA; 13 University of Utah, Salt Lake City, UT USA; 14 Children’s National Hospital, The George Washington University, Washington, D.C USA; 15 University of Washington, Seattle, WA USA

**Keywords:** Large language models, Sentiment analysis, Emergency medical services, Pediatrics, Research ethics, Medical ethics, Paediatrics, Public health

## Abstract

Large language models (LLMs) can improve text analysis efficiency in healthcare. This study explores the application of LLMs to analyze patient perspectives within the exception from informed consent (EFIC) process, which waives consent in emergency research. Our objective is to assess whether LLMs can analyze patient perspectives in EFIC interviews with performance comparable to human reviewers. We analyzed 102 EFIC community interviews from 9 sites, each with 46 questions, as part of the Pediatric Dose Optimization for Seizures in Emergency Medical Services study. We evaluated 5 LLMs, including GPT-4, to assess sentiment polarity on a 5-point scale and classify responses into predefined thematic classes. Three human reviewers conducted parallel analyses, with agreement measured by Cohen’s Kappa and classification accuracy. Polarity scores between LLM and human reviewers showed substantial agreement (Cohen’s kappa: 0.69, 95% CI 0.61–0.76), with major discrepancies in only 4.7% of responses. LLM achieved high thematic classification accuracy (0.868, 95% CI 0.853–0.881), comparable to inter-rater agreement among human reviewers (0.867, 95% CI 0.836–0.901). LLMs enabled large-scale visual analysis, comparing response statistics across sites, questions, and classes. LLMs efficiently analyzed patient perspectives in EFIC interviews, showing substantial sentiment assessment and thematic classification performance. However, occasional underperformance suggests LLMs should complement, not replace, human judgment. Future work should evaluate LLM integration in EFIC to enhance efficiency, reduce subjectivity, and support accurate patient perspective analysis.

**Supplementary Information:**

The online version contains supplementary material available at 10.1038/s41598-025-89996-w.

## Background & significance

Large language models (LLMs) have emerged as powerful tools for generating, summarizing, and analyzing complex text with natural language understanding^1–3^. Recently, LLMs have been applied in healthcare, enhancing tools such as diagnostics, decision-making support, and predictive analytics^4^. While these applications have garnered considerable interest, the challenge of using LLMs to summarize and understand patient perspectives has received less attention despite its critical importance^5,6^. This involves analyzing and aggregating large volumes of patient narratives, a task for which LLMs are well-suited.

We explore how LLMs can be applied to understand patient perspectives in healthcare, using the exception from informed consent (EFIC) process as a use case. The EFIC process allows the enrollment of subjects in clinical trials when consent isn’t feasible, e.g., for administering an automated external defibrillator to unconscious patients^7^. Instead of obtaining consent directly from the patient, EFIC requires community-wide interviews before starting a trial^9^. Analyzing these interviews presents a major challenge, requiring extensive manual labor and subjective judgment with ambiguous standardization^8–11^.

## Objective

This challenge presents an opportunity for LLMs to efficiently analyze and interpret patient perspectives, addressing issues of scale and consistency. In this study, we hypothesized that LLMs would match human reviewers in determining patient perspectives, including sentiment (e.g., very positive to very negative), and in classifying responses from EFIC interviews into themes. We tested this hypothesis using LLMs to analyze sentiment polarity and classify interviews from the Pediatric Dose Optimization for Seizures in Emergency Medical Services (PediDOSE) study, comparing their performance with human reviewers.

## Results

### LLM polarity score analysis

**Fig. 1:**
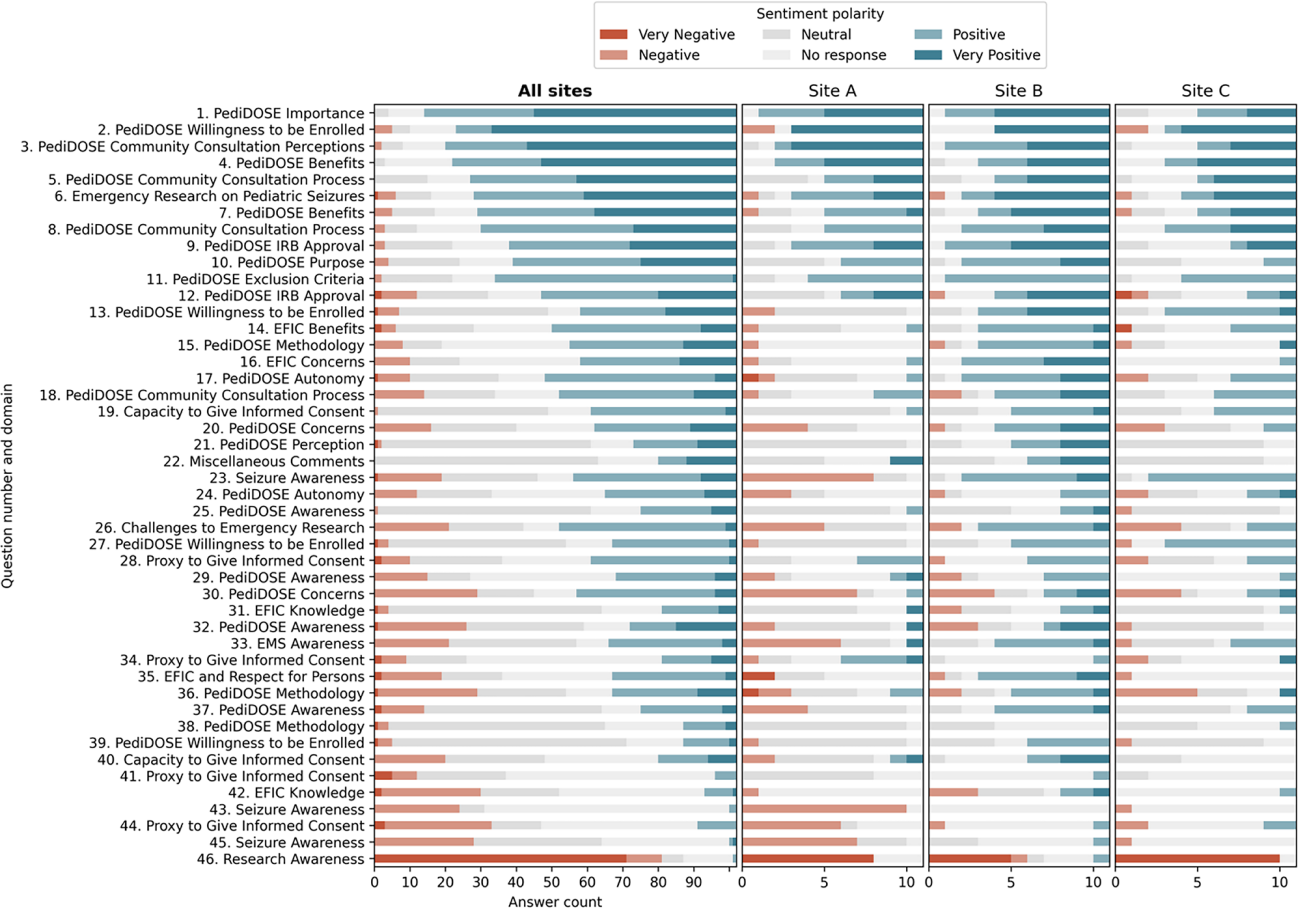
Large language models (LLM) polarity score across participating sites (*n* = 3,692 responses). Each row represents the mean LLM response polarity score to each question, color indicates assigned polarity. Each question is represented by question number and representative domain. *All sites* represent the mean LLM response polarity score from all nine sites, while the subsequent three columns provide a breakdown of the response polarity score at three sites, selected for detailed comparison to simplify review.

We first used GPT-4 to assign polarity scores to interview responses across the study questions (Fig. 1**).** Of 3,692 responses available for analysis, 1,000 were coded “no response.” GPT-4 classified 2.8% (*n* = 104) of all site responses as very negative, 13.1% (*n* = 482) as negative, 32.7% (*n* = 1207) as neutral, 32.3% (*n* = 1191) as positive, and 19.2% (*n* = 708) as very positive. Additionally, visualizing sentiment polarity across sites generally revealed consistent trends in responses, while also highlighting specific questions that differed between sites. For example, Question 32 on seizure awareness elicited more negative responses from Site A compared to the other two sites.

**Fig. 2:**
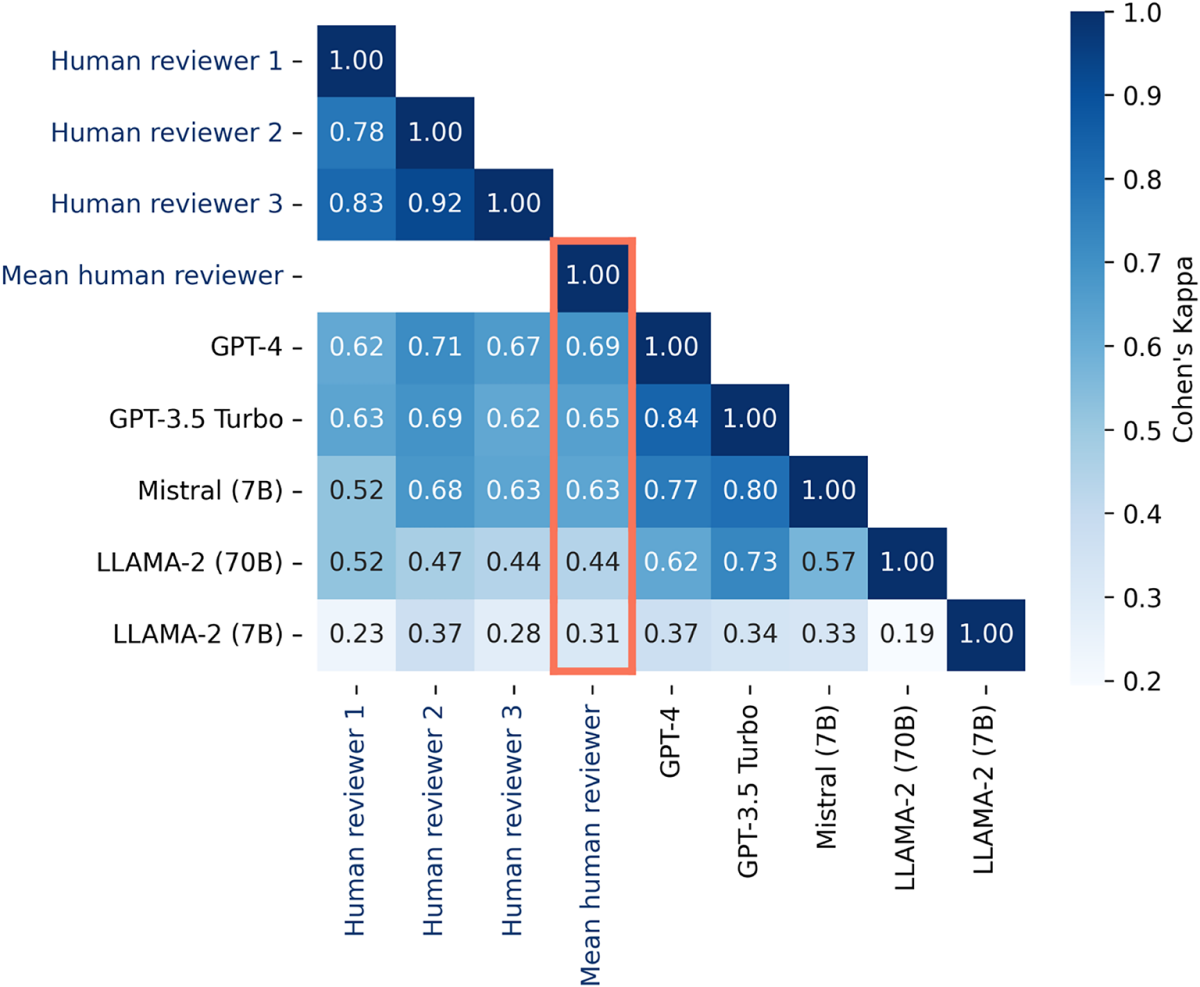
Polarity score agreement between mean, individual human reviewers, and large language models (LLM) across human reviewed sample (*n* = 123 responses). Using Cohen’s Kappa, this heatmap shows agreement of response polarity scores between human reviewers and LLM classes.

To compare LLM-generated judgments to human judgments, 5 LLMs and 3 human reviewers assigned polarity scores to 123 responses to 9 questions from 3 PediDOSE sites (Fig. 2**).** The mean human reviewer polarity score substantially agreed with GPT-4, the highest-performing LLM of the five LLMs evaluated, yielding a Cohen’s kappa k = 0.69 (95% CI 0.61–0.76), Fig. 2. In comparison, individual human reviewer polarity scores yielded a slightly higher agreement among the 3 reviewers, ranging from k = 0.78–0.92. Mistral (7B) k = 0.63, GPT-3.5 Turbo k = 0.65, and GPT-4 k = 0.69 substantially agreed with mean human reviewer polarity score. The highest performing LLMs (GPT-4, GPT 3.5 turbo, Mistral) also had the most substantial agreement with human reviewer 2. LLAMA 2 (7B), k = 0.31 and LLAMA (70B) k = 0.44 had the lowest agreement with the mean human reviewer polarity score. The lowest performing LLMs (LLAMA (7B) and (70B)) had very poor agreement with each other, k = 0.19.

Major discrepancies, in which the GPT-4 and the human reviewer assigned opposite polarity scores (e.g. positive vs. negative), were seen in 4.7% of all scores. Human reviewers were 62% less likely to assign extreme values (very positive and very negative) than GPT-4. Most questions that generated a positive polarity score from LLM were also scored as positive by the human reviewers. For example, LLM and human reviewers scored this question as positive, Question 1, “How important do you think it is to do this study in your community?” **(Table A1**,** Supplementary File 1)**. Similarly, questions that yielded a negative polarity score from LLM we also scored as negative by the human reviewers. Most negative polarity score questions were about the background or are phrased in a manner that they expect negative responses, e.g., Question 46 “Do you have any remaining questions about research or informed consent?” A common answer is “nothing else”.

## Thematic classification

**Fig. 3:**
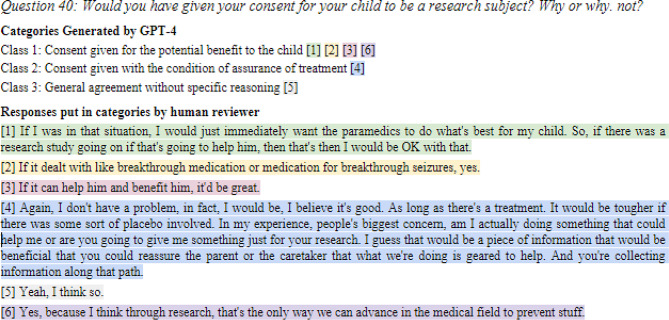
Thematic classification by human reviewers. Human reviewers classified the responses to Question 40 into GPT-4 generated classes.

Up to 15 responses for each question were randomly selected from three study sites. In our text classification analysis of GPT-4, we collected 188 responses from 22 free-text questions sorted into classes by each human reviewer; an example is shown in Fig. 3. On average, GPT-4 generated 3.24 classes per question. Human reviewers categorized responses into the same classes as GPT-4 86.8% (95% CI 86.3–87.3%) of the time, suggesting that thematic classification by GPT-4 is similar to that by human reviewers (Table 1). Inter-reviewer thematic classification accuracy was 86.7% (95% CI 85.3–88.1%).

Thematic classification accuracy of response classes by human review compared to GPT-4, inter-reviewer agreement (% accuracy ± 95% confidence interval).

**Table 1:** Text classification accuracy.

Human Reviewer-GPT-4 Accuracy *n* = 188 responses			Human Reviewer-Human Reviewer Accuracy
Reviewer 1	Reviewer 2	Reviewer 3	Inter-reviewer agreement
86.1 ± 4.9%	86.7 ± 4.9%	87.7 ± 4.7%	86.7% ±2.7%

## Discussion

Our study demonstrates that LLMs assigned sentiment polarity scores and classified responses from EFIC community interviews for PediDOSE nearly as well as human reviewers. However, LLMs had less agreement in assessing polarity scores compared to human reviewers, highlighting the need for LLMs to complement, rather than replace, human oversight. By combining human and LLM evaluations, we achieve a more comprehensive quantitative analysis, which enhances our understanding of patient sentiment. LLMs can also provide clear, concise class-based summaries, enabling investigators, Institutional Review Board (IRB), and ethics boards to rapidly assess overall responses. Sentiment polarity scores offer a quantitative assessment of community sentiment, helping stakeholders understand not only overall trends but also variations in participant responses to individual questions, which can inform targeted improvements in communication and study design. Quantitative plots generated from LLM data, such as those in Fig. 1, facilitate the rapid analysis of large datasets, enabling visual interpretation of patterns, including variations and outliers. Our study showed that responses to the same question across 3 sites were generally consistent, with few exceptions. These plots helped identify trends and outliers between sites. For example, responses to Question 23 (personal experiences with seizures) at Site A showed significantly more negative sentiment compared to the other two sites. Such insights help understand patient sentiment nuances, enhancing overall analysis.

LLMs were more likely than human reviewers to assign extreme polarity values. One explanation is that LLMs may struggle to recognize the subtleties of human language. Another possibility is that the model’s performance metric, designed to reward higher confidence, could bias polarity scoring toward extremes. Additionally, LLMs may struggle to interpret emotional context, which can result in more extreme classifications based on the text’s surface content rather than its deeper emotional nuances^12^. This underscores the need for sentiment polarity assessments to be considered alongside thematic classification for more accurate interpretation.

Our thematic classification process improved our understanding of community feedback and may assist Food and Drug Administration (FDA) EFIC procedures related to study protocol approvals, adjustments, or disapprovals. Classifying responses into distinct classes provides researchers and regulatory bodies with deeper insights into community sentiments, facilitating more comprehensive evaluations of clinical studies. Thematic classification has been applied across various fields, including healthcare. For instance, non-generative LLMs have classified patients into health categories from discharge summaries^13^. In our study, Question 40 highlighted a range of community opinions about children’s participation in research, emphasizing the requisite for clear and transparent communication about the EFIC process to future participants.

Classifying large volumes of lengthy responses is a complex, time- and resource-intensive process. In our study, LLMs performed similarly to human reviewers, offering valuable support in enhancing the efficiency of thematic classification, particularly with large datasets. Our findings demonstrate that LLMs can assist in assigning responses to predefined thematic classes, a key step in the EFIC process that traditionally requires significant human effort. This capability enables investigators and ethics boards to rapidly summarize and interpret feedback, potentially streamlining future community consultation workflows and improving the consistency of data interpretation. Through prompt engineering, LLMs generate classes and organize responses under these classes with corresponding citations. This method helps indicate which responses align with each class. LLMs greatly facilitated the work of human reviewers, enabling quick assessment of class validity, while reviewers can easily verify the accuracy of classifications by referencing source responses.

LLMs did not match humans in analyzing patient perspectives, but the field is advancing rapidly. Improvements in modeling^14,15^, prompting^16,17^, and interpretation^18^will enhance sentiment analysis. “Hallucinations,” or fabricated information generated by LLMs, remain a challenge^19,20^. Investigators are exploring innovative methods for LLMs to detect these issues, such as self-verification^19^. Similarly, as LLMs’ context windows grow, they’ll handle larger datasets, improving classification of lengthy interviews.

Further studies are needed to assess whether human reviewers can adjust and refine LLM-generated classifications to align with nuanced human judgments. This approach leverages the strengths of both LLMs and human oversight for more reliable and accurate text classification. One study noted that analyzing survey data for the FDA is challenging due to a lack of standardized formats and raw data^21^. Our findings suggest that using LLMs can improve the efficiency of analyzing community consultation data for EFIC trials and help standardize reporting, building trust for future studies. Although this study did not measure workflow metrics, such as the time required to create summary reports or IRB review turnaround times, the visual representation of quantified sentiment and thematic classifications illustrates how LLM-generated outputs can streamline regulatory processes. For example, LLM-generated sentiment polarity plots could help IRBs and ethics committees quickly identify trends, such as a notable percentage of participants raising concerns about consent processes or reporting confusion about study procedures. This approach reduces the cognitive burden of manually reviewing extensive unstructured data and enables more efficient, data-driven decision-making. Future studies should consider quantifying time savings and evaluating user feedback to further demonstrate the practical impact of LLMs in regulatory workflows.

## Limitations

Our study has several limitations. First, we limited our analysis to a manageable number of human reviews. Manually reviewing and annotating interviews is labor-intensive, underscoring the challenge of the EFIC process and the potential utility of LLMs in facilitating those tasks. Regardless, we conducted ample human annotations to reach reliability and meaningful correlation. Second, we limited our human reviewers to investigators. Future studies could include other EFIC team members or other stakeholders, such as IRB members, to review and modify the data output to suit their needs. Third, although LLMs do not need to analyze yes/no responses, we included all question types for sentiment polarity assessment to demonstrate the full range of LLM capabilities and to avoid introducing bias through selective exclusion. While some questions may seem binary, participant responses often included elaborations that provided valuable sentiment insights. By including all questions, we ensured that the analysis captured these nuances and demonstrated the LLM’s ability to handle a wide range of input formats. This approach allows future users to tailor their analyses to specific research needs while maintaining transparency in how LLMs can be applied. Fourth, our study design limited our ability to investigate how demographics such as race, ethnicity, language, and other patient-level experiences influence the responses to the interview questions. Fifth, presenting the survey questions in a set order may have introduced a bias based on sequence. However, this approach reflects how surveys are done in the real world, offering an authentic portrayal of EFIC activities. Similarly, to mitigate bias and ensure that each response from the LLM was generated solely based on the text of that response, without undue influence from prior responses, the interface was systematically reset after each query. Sixth, another limitation of our study is the absence of demographic data linked to individual interview responses, which prevented a detailed analysis of LLM performance across different population subgroups. Demographic factors such as language proficiency, cultural norms, and socio-economic background may influence response patterns and LLM classifications. Future studies should consider integrating demographic information to explore whether LLMs perform differently across subgroups and to detect potential biases in sentiment classification. Addressing these biases is essential to ensuring that LLM-based approaches in healthcare research remain equitable and reflective of diverse community perspectives.

## Conclusion

Overall, LLMs demonstrated substantial agreement with human reviewers in analyzing patient perspectives, including sentiment polarity and thematic classification, using EFIC community interviews. While LLM polarity scoring showed lower reliability compared to human reviewers, thematic classification performance closely matched human assessments. Our study emphasizes the importance of using LLMs to supplement, rather than replace, human oversight. LLMs provide an effective method for rapidly summarizing and visualizing large datasets. Future efforts should explore how stakeholders can leverage LLMs to deepen insights into patient perspectives across various healthcare settings.

## Materials and methods

### Setting

PediDOSE is a multicenter EFIC trial (NCT05121324) that seeks to decrease the number of children who continue to have seizures upon arrival in the emergency department. Specifically, it evaluates the effectiveness of a standardized emergency medical services (EMS) protocol with age-based, paramedic-administered midazolam dosing^22^.

Following the EFIC process, PediDOSE sites conducted EFIC community interviews to obtain IRB approval. Specifically, 10–14 community consultation interviews were conducted across 20 centers using an interview guide composed of 46 questions **(Supplement Table A1**,** Supplementary File 1).**^23^ These questions were grouped into 25 domains preselected by EFIC PediDOSE investigators (Fig. 1). Interviewers were allowed to skip questions that seemed irrelevant based on previous responses from the interviewee and to ask unscripted follow-up questions. Interviews were conducted in English or Spanish, recorded, translated into English by certified interpreters, and de-identified for local IRB and investigator approval. Each site archived and transcribed interviews, which were submitted to the central IRB for review. All PediDOSE EFIC interview data were collated for manual review and final EFIC approval.

### Study design

**Fig. 4:**
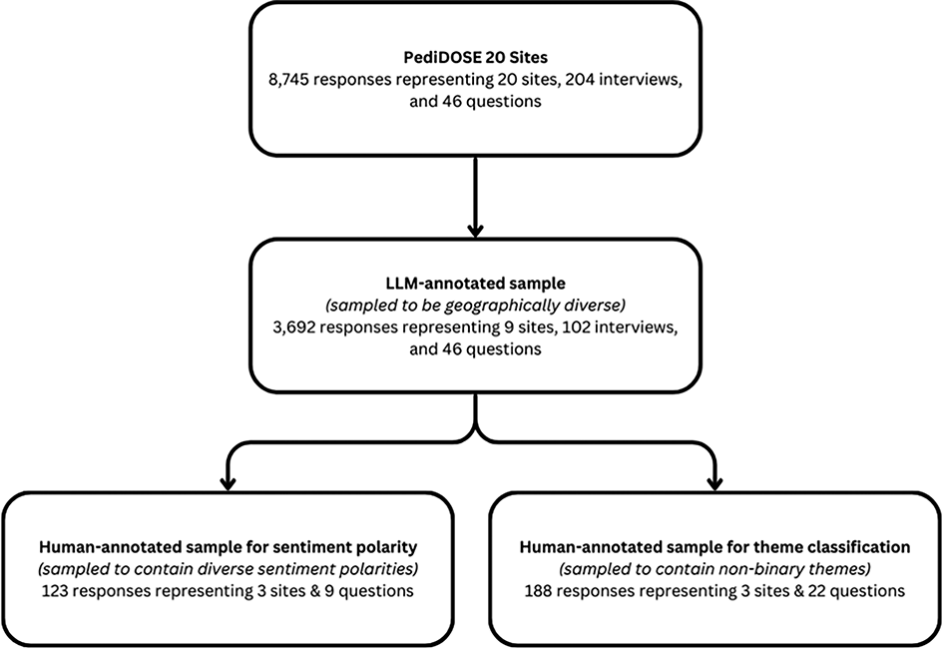
Sampling Data Flow of Interview Responses. A visual overview of the data flow, showing the original dataset of Exception From Informed Consent (EFIC) community interviews collected across 20 Pediatric Dose Optimization for Seizures in Emergency Medical Services (PediDOSE) study sites, the geographically diverse subset annotated by large language models (9 sites), and the final human-annotated samples used for sentiment polarity and thematic classification comparisons.

We retrospectively reviewed interview transcripts from the EFIC community interviews in the PediDOSE study. This study was approved by the central IRB, the University of Utah, and all participating site IRBs. All methods were performed in accordance with relevant guidelines and regulations, including the Declaration of Helsinki. Written informed consent was obtained from all interview participants, as applicable. Identifiable information was removed to ensure participant anonymity. All actions were part of the PediDOSE EFIC activities, except for the post-hoc analysis of the transcripts performed as part of this study. For our analysis, we selected nine geographically diverse PediDOSE study sites that were representative of the various regions and patient demographics under study (Fig. 4). The University of Utah approved a waiver of written informed consent to collect and analyze the interview recordings; no identifiable information was requested during the recorded and transcribed interview. The source code used for performing the analyses is available at https://github.com/csinva/pedidose-efic-analysis. The study followed the Strengthening the Reporting of Observational Studies in Epidemiology (STROBE) reporting guidelines and the JAMA Network Guidance for evaluating and reporting LLM research^24–26^.

### Analysis methods

We evaluated two closed-source LLMs: GPT-4 (gpt-4-0613, Open AI)[17] and GPT-3.5 (gpt-3.5-turbo-0613, Open AI)^27^, which were both accessed securely through the Azure OpenAI Application Programming Interface (API). We additionally assessed three open-source models: Mistral 7B (Mistral AI), LLaMA-2-7B (Meta), and LLaMA-2-70B (Meta), which were run locally for secure HIPAA-compliant computing. To minimize randomness, each LLM query was run in an independent session with a sampling temperature of 0. Python version 3.11 (Python Software Foundation) was used for all statistical analyses.

### LLM-based sentiment polarity analysis

We evaluated each LLM’s ability to assess the sentiment polarity for all responses in the studied interviews. Specifically, an LLM was prompted to categorize the polarity of a response on a 5-point Likert scale (very positive, positive, neutral, negative, or very negative*)*. For example, in response to a question such as *Is this study important to you?*, answers can vary from *very positive* (indicating it is important) to *very negative* (suggesting it is not important). If a question was not asked or if the respondent did not respond directly, it was coded as *no response*.

### LLM-based thematic classification

In our theme classification analysis, we used two-step prompts to categorize participant responses. The first prompt listed the 25 predefined classes relevant to the EFIC process (e.g., “concerns about consent,” “clarity of study purpose”) and asked GPT-4 to sort responses into one of these classes. The second prompt aggregated responses within each class to generate a comprehensive summary of participant feedback. The detailed prompt structure can be found in **Table A2**,** Supplementary File 1**.

### Human reviewer assessments

We evaluated the LLM-based sentiment polarity analysis and the LLM-based thematic classification by comparing LLM assessments to those of 3 blinded human reviewers. Human reviewers were made to do the same two analyses as the LLM. To allocate human reviewer time effectively, we subsampled each analysis’s responses. For sentiment polarity analysis, we selected 15 responses from 9 questions across 3 random sites, stratified to yield a uniform range of negative, neutral, and positive answers. For thematic classification, we excluded simple ‘Yes’ or ‘No’ responses, as they do not require LLM analysis and would not add complexity to the problem. In cases of disagreement among reviewers, the majority opinion was used for analysis.

### Evaluation metrics

We hypothesized that there would be a substantial association between the LLMs’ outputs and human reviewers. For sentiment polarity scores, we measure the association using Cohen’s Kappa k. For thematic classification, we measure the association using classification accuracy. Then, we use the LLMs scores to generate quantitative plots that enable understanding sentiment polarity distributions across sites and questions.

## Electronic supplementary material

Below is the link to the electronic supplementary material.

Supplementary Material 1

## Data Availability

Partial datasets and data dictionaries for the parent investigation, Pediatric Dose Optimization for Seizures in Emergency Medical Services Study (PediDOSE), will be available from the Data Coordinating Center (DCC) of the Pediatric Emergency Care Applied Research Network (PECARN) in a de-identified format 3 years after the last participant enrollment (anticipated July 2029). However, the qualitative transcripts of this secondary analysis cannot be made available because they cannot be de-identified. Please contact Dr. Manish Shah, mshah5@stanford.edu.

## Abbreviations

API: Application Programming Interface
EFIC: Exception From Informed Consent
EMS: Emergency Medical Services
FDA: Food and Drug Administration
IRB: Institutional Review Board
LLMs: Large Language Models
PediDOSE: Pediatric Dose Optimization for Seizures in Emergency Medical Services
STROBE: Strengthening the Reporting of Observational Studies in Epidemiology
