# Supplementary material for: Analyzing patient perspectives with large language models: a cross-sectional study of sentiment and thematic classification on exception from informed consent
Source: Sci Rep. 2025 Feb 20;15:6179. doi: 10.1038/s41598-025-89996-w (PMC11842787; doi:10.1038/s41598-025-89996-w)
Supplement: Supplementary file 1 — Supplementary Material 1 [file 41598_2025_89996_MOESM1_ESM.pdf]

Article Title: Analyzing Patient Perspectives with Large Language Models: A Cross-Sectional Study on Exception from Informed Consent

Journal Name: Journal of Healthcare Informatics Research

Author Names & Affiliation:
Aaron E. Kornblith, MD*
Associate Professor of Emergency Medicine & Pediatrics
University of California, San Francisco, San Francisco, CA, USA

Chandan Singh, PhD (co-first)
Microsoft Research
Affiliate of the Department of Emergency Medicine
University of California, San Francisco, San Francisco, CA, USA

Johanna C. Innes, MD, NRP
Assistant Professor of Emergency Medicine
Jacobs School of Medicine and Biomedical Sciences, University at Buffalo, Buffalo, NY, USA

Todd P. Chang, MD, MAcM
Associate Professor of Pediatrics & Medical Education
Keck School of Medicine of University of Southern California & Children's Hospital Los Angeles, Los Angeles, CA, USA

Kathleen M. Adelgais, MD MPH
Professor, Pediatrics and Emergency Medicine
University of Colorado School of Medicine, Aurora, CO, USA

Maija Holsti, MD, MPH
Professor of Pediatrics
Primary Children's Medical Center, University of Utah, Salt Lake City, UT, USA

Joy Kim, BS, CCRP
Senior Clinical Research Assistant
Oregon Health & Science University, Portland, OR, USA

Bradford McClain, MA
Clinical Research Coordinator
Cincinnati Children's Hospital Medical Center, Cincinnati, OH, USA

Daniel K. Nishijima, MD, MAS
Professor of Emergency Medicine
UC Davis School of Medicine, Sacramento, CA, USA

Steffanie Rodgers, BS
EMS/Trauma Clinical Research Coordinator Team Lead

Nationwide Children’s Hospital, Columbus, OH, USA

Manish I. Shah, MD, MS
Professor of Emergency Medicine
Stanford University School of Medicine, Palo Alto, CA, USA

Harold K. Simon, MD, MBA
Professor of Pediatrics and Emergency Medicine
Emory University School of Medicine & Children’s Healthcare of Atlanta, Atlanta, GA, USA

John M. VanBuren, PhD
Associate Professor
Department of Pediatrics, University of Utah, Salt Lake City, UT, USA

Caleb E. Ward MB BChir, MPH
Assistant Professor of Pediatrics and Emergency Medicine
Children’s National Hospital; The George Washington University School of Medicine and Health Sciences, Washington D.C., USA

Catherine R. Counts, PhD, MHA
Acting Assistant Professor
Department of Emergency Medicine, University of Washington, Seattle, WA, USA

***Corresponding Author**
Aaron Kornblith, MD
550 16th Street, Box 0649
San Francisco CA 94143

E-mail of Corresponding Author: Aaron.Kornblith@ucsf.edu

SUPPLEMENT

To evaluate if an LLM could assign responses to human-generated classes, first, we prompted the LLM to sort the responses into human-generated classes. We then determined whether the number of responses assigned to each class by the LLM matched that of the reviewer (**Fig A1**). Note that the human-reviewer class never has a count of 0. Questions for which there was only one human-generated class or for which the number of responses in classes did not match the number of responses were excluded, resulting in 261 questions across three sites. The accuracy (i.e., percentage of assignments that agreed with reviewer) was 41.3% for GPT-4 and 36.8% for GPT-3.5 Turbo.

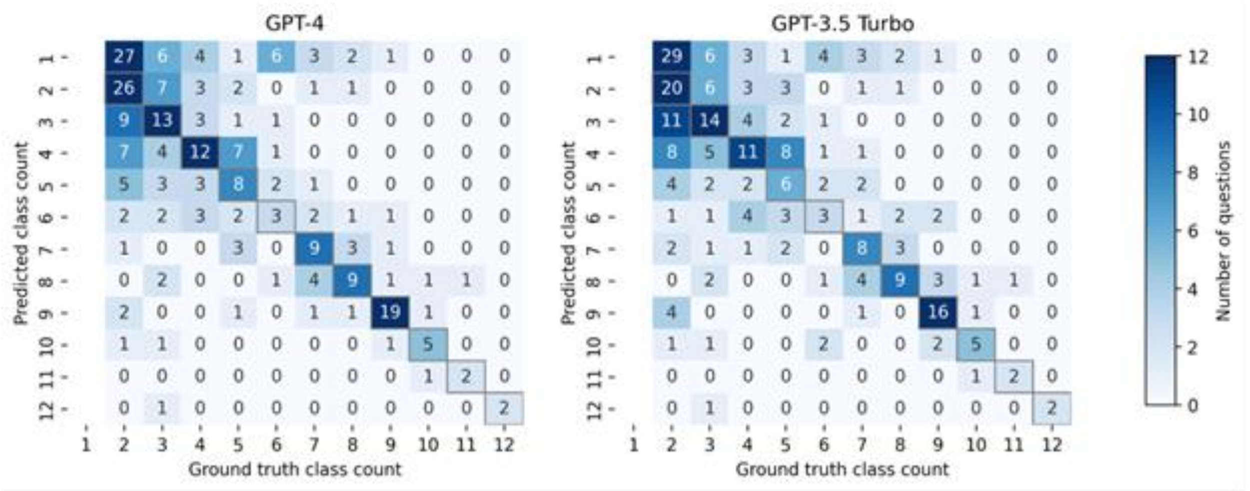

Fig A1. GPT-4 text classification performance of responses into human-generated classes (Ground truth class). Performance was measured by whether the number of responses assigned to each class by the LLM matched that of a human reviewer.

Table A1. PediDOSE community consultation interview questions and assigned domains.

Assigned Domain	Question
PediDOSE importance	1. How important do you think it is to do this study in your community?
PediDOSE willingness to be Enrolled	2. If this study did not involve an emergency, and we had time to consent you before treatment, what would be your decision to have your child participate?
PediDOSE community consultation Perceptions	3. Who do you think should be included in this community consultation process?
PediDOSE benefits	4. Who do you think would possibly benefit from this study?
PediDOSE community consultation process	5. How would you prefer to receive information about studies like this?
Emergency research on pediatric seizures	6. What are your thoughts about research trials that test medications to treat seizures?
PediDOSE benefits	7. How do you think this study might improve medical care for children with seizures?
PediDOSE community consultation process	8. What media or methods should be used to inform the community about the PediDOSE study?
PediDOSE IRB approval	9. If your child had a seizure and was included in this study, who would you have wanted the study team to talk to before this study was approved by the board?
PediDOSE purpose	10. What is your understanding about this study's purpose?
PediDOSE exclusion criteria	11. Is there any reason, other than the qualifying criteria we have gone over, that you think someone should not be able to be enrolled without their permission?
PediDOSE IRB approval	12. Who should the study team need to hear from before presenting this study to the institutional review board that approves studies like this?
PediDOSE willingness to be enrolled	13. A minor CHILD/family member is having a seizure when the paramedics arrived to your home. He/she meets the qualifications to participate in the PediDOSE study. Would you agree to have him or her being included in the research study without your permission or your family's permission ahead of time?
EFIC benefits	14. What do you think are the benefits of doing research in emergency situations without a patient's informed consent?

PediDOSE methodology	15. What concerns, if any, do you have about the random assignment of the ambulance agencies regarding when they switch to the new method of dosing?
EFIC concerns	16. What concerns do you have about starting research-related treatments in an emergency situation if you could not provide consent for yourself or on behalf of your child?
PediDOSE autonomy	17. How do you feel about not having a choice about participation in the study?
PediDOSE community consultation process	18. If community consultation were not feasible, what other methods do you think we should use to gather feedback on how best to do emergency research like this?
Capacity to give informed consent	19. In that moment, when a paramedic is getting ready to treat your child who is having a seizure, would you have been able to make an informed decision about whether or not to participate in a research study?
PediDOSE concerns	20. What are your fears or worries, if any, regarding the study?
PediDOSE perception	21. Based on what you just heard, do you have any questions about WHAT we plan to do in this study?
Miscellaneous comments	22. Would you like to share any final comments or suggestions before I end the recording?
Seizure awareness	23. Tell me a little about your experience with seizures
PediDOSE autonomy	24. Why do you feel that way, and are those reasons related to prior experiences you or others have had?
PediDOSE awareness	25. Do you have any questions after watching the video?
Challenges to emergency research	26. What do you think could be some of the challenges when doing research on emergency, time-sensitive conditions?
PediDOSE willingness to be enrolled	27. If this PediDOSE study enrolled ADULTS and then adult family member was having a seizure and he or she met the qualifications to participate in the study, would you agree if it was an adult in being consented or being enrolled without permission ahead of time?
Proxy to give informed consent	28. Would you want somebody else to decide if your child could be a research subject? If so, who?
PediDOSE awareness	29. What questions do you have about the study?
PediDOSE concerns	30. What, if anything, is there that could change regarding the details of this study to make you feel comfortable with being enrolled without your permission?
EFIC knowledge	31. Do you have any questions about these regulations for emergency research?

PediDOSE awareness	32. What have you heard about the study so far that did not make sense?
EMS Awareness	33. Tell me about a time when you have had to call 9-1-1…?
Proxy to give informed consent	34. Who would you want to make that decision, if you were unable and a family member or close friend (or other proxy mentioned) were not available in time?
EFIC and respect for persons	35. What do you think researchers could do to promote respect for a patient's decision making without getting informed consent?
PediDOSE methodology	36. What is your understanding about why the ambulance agencies will be randomly assigned to when they will switch methods of dose determination?
PediDOSE awareness	37. What had you heard about this study before today?
PediDOSE methodology	38. Do you have any questions about HOW we plan to do this study...?
PediDOSE willingness to be enrolled	39. If PediDOSE enrolled adults and YOU are having a seizure when paramedics arrived at your home, assuming you meet the study qualifications to participate, would you agree to be included in the research study, without your permission?
Capacity to give informed consent	40. Would you have given your consent for your child to be a research subject? Why or why not?
Proxy to give informed consent	41. Would you want someone else besides a family member or close friend (or other proxy mentioned) to decide if your child could be a research subject?
EFIC knowledge	42. What is your knowledge about the U.S. government's regulations about doing emergency research without asking for consent ahead of time?
Seizure awareness	43. What was your or your child's experience like?
Proxy to give informed consent	44. What if a family member or close friend (or other proxy mentioned) was not available in time?
Seizure awareness	45. Have you or a family member ever had a seizure?
Research awareness	46. Questions about research or informed consent

Table A2. Prompt details

Prompt for sentiment polarity rating:

‘‘‘### You are given a question and a response. Rate the sentiment/supportiveness of the response on a scale of 1 to 5, where 1 is very negative and 5 is very positive. ###

Question: {question}

Response: {response}

Rating (1-5):’’”

Prompt for text classification:

‘‘‘### You are given a question, response, and a numbered list of themes below.

Question: {question}

Response: {response}

Themes:
{classes_as_numbered_list}

Which of the themes does the response above belong to? Return the theme number.

Answer:’’’

Prompt for class generation:

‘‘‘### You are given a question and a set of responses below.

Question: {question}

Responses:
{response_list}

Group all responses into 2 or more nonoverlapping themes.

Return a comma-separated list, where each element is a theme, followed by the numbers of the responses that fall into that theme in brackets.

Example answer: Theme 1: Negative responses [1, 2, 5], Theme 2: Positive responses [3, 4]

Answer: Theme 1:’’’

Pediatric Emergency Care Applied Research Network (PECARN) Pediatric Dose Optimization for Seizures in EMS (PediDOSE) Study Investigators

(in alphabetical order by last name)

1. Kathleen M. Adelgais, MD, MPH
2. Nichole Bosson, MD
3. Kathleen M. Brown, MD
4. Lorin R. Browne, DO
5. James M. Chamberlain, MD
6. Todd P. Chang, MD, MAcM
7. Brian M. Clemency, DO, MBA
8. Cinnamon A. Dixon, DO, MPH
9. Gregory W. Faris, MD
10. Raymond L. Fowler, MD
11. Joshua B. Gaither, MD
12. Marianne Gausche-Hill, MD
13. Nancy K. Glober, MD
14. Nicolaus W. Glomb, MD, MPH
15. Matthew L. Hansen, MD, MCR
16. Adam L. Hartman, MD
17. Maija Holsti, MD, MPH
18. Johanna C. Innes, MD
19. Kathryn M. Kothari, MD
20. Lekshmi Kumar, MD
21. Andrew J. Latimer, MD
22. Julie C. Leonard, MD, MPH
23. E. Brooke Lerner, PhD
24. Geoffrey S. Lowe, MD
25. Christian Martin-Gill, MD, MPH
26. Lindsey A. Morgan, MD
27. Claudia R. Morris, MD
28. Daniel K. Nishijima, MD, MAS
29. Stacey K. Noel, MD
30. Daniel G. Ostermayer, MD
31. Sylvia Owusu-Ansah, MD, MPH
32. Amber D. Rice, MD
33. Lauren C. Riney, DO
34. James J. Riviello, MD
35. Rana R. Said, MD
36. Mohsen Saidinejad, MD, MS, MBA
37. Kevin J. Schulz, MD
38. Manish I. Shah, MD, MS
39. Robert Silbergleit, MD
40. Harold Simon, MD

41. Daniel W. Spaite, MD
42. Jonathan R. Studnek, PhD, NRP
43. Joseph E. Sullivan, MD
44. Douglas R. Swanson, MD
45. Neil G. Uspal, MD
46. John M. VanBuren, PhD
47. Henry E. Wang, MD, MS, MPH
48. Caleb E. Ward, MB BChir, MPH
49. Benjamin W. Weston, MD, MPH
50. Denise A. Whitfield, MD, MBA
51. Scott T. Youngquist, MD, MS
